# Primary Cultures of Glomerular Parietal Epithelial Cells or Podocytes with Proven Origin

**DOI:** 10.1371/journal.pone.0034907

**Published:** 2012-04-18

**Authors:** Nazanin Kabgani, Tamara Grigoleit, Kevin Schulte, Antonio Sechi, Sibille Sauer-Lehnen, Carmen Tag, Peter Boor, Christoph Kuppe, Gregor Warsow, Sandra Schordan, Jörg Mostertz, Ravi Kumar Chilukoti, Georg Homuth, Nicole Endlich, Frank Tacke, Ralf Weiskirchen, Georg Fuellen, Karlhans Endlich, Jürgen Floege, Bart Smeets, Marcus J. Moeller

**Affiliations:** 1 Division of Nephrology and Immunology, University Hospital of the Aachen University of Technology (RWTH), Aachen, Germany; 2 Department of Cell Biology, University Hospital of the Aachen University of Technology (RWTH), Aachen, Germany; 3 Division of Gastroenterology, University Hospital of the Aachen University of Technology (RWTH), Aachen, Germany; 4 Institute of Clinical Chemistry and Pathobiochemistry, Comenius University, Bratislava, Slovakia; 5 Institute of Pathology and Institute of Molecular Biomedicine, Comenius University, Bratislava, Slovakia; 6 University Hospital of the Aachen University of Technology (RWTH), Aachen, Germany; 7 Institute for Biostatistics and Informatics in Medicine and Ageing Research, University of Rostock, Rostock, Germany; 8 Department of Mathematics and Informatics, Ernst Moritz Arndt University Greifswald, Greifswald, Germany; 9 Department of Anatomy and Cell Biology, Ernst Moritz Arndt University Medicine Greifswald, Greifswald, Germany; 10 Interfaculty Institute of Genetics, Department of Functional Genomics, Ernst Moritz Arndt University, Greifswald, Germany; University of Jaén, Spain

## Abstract

Parietal epithelial cells (PECs) are crucially involved in the pathogenesis of rapidly progressive glomerulonephritis (RPGN) as well as in focal and segmental glomerulosclerosis (FSGS). In this study, transgenic mouse lines were used to isolate pure, genetically tagged primary cultures of PECs or podocytes using FACsorting. By this approach, the morphology of primary glomerular epithelial cells in culture could be resolved: Primary podocytes formed either large cells with intracytoplasmatic extensions or smaller spindle shaped cells, depending on specific culture conditions. Primary PECs were small and exhibited a spindle-shaped or polygonal morphology. In the very early phases of primary culture, rapid changes in gene expression (e.g. of WT-1 and Pax-2) were observed. However, after prolonged culture primary PECs and podocytes still segregated clearly in a transcriptome analysis - demonstrating that the origin of primary cell cultures is important. Of the classical markers, synaptopodin and podoplanin expression were differentially regulated the most in primary PEC and podocyte cultures. However, no expression of any endogenous gene allowed to differentiate between the two cell types in culture. Finally, we show that the transcription factor WT1 is also expressed by PECs. In summary, genetic tagging of PECs and podocytes is a novel and necessary tool to derive pure primary cultures with proven origin. These cultures will be a powerful tool for the emerging field of parietal epithelial cell biology.

## Introduction

In recent years, major advances in our understanding of the function and biology of glomerular parietal epithelial cells (PECs) have been made. In particular, PECs play a major role in glomerular physiology and diseases. First, it has been shown that in murine kidney development about 10% of the podocytes are recruited from PECs and/or transitional cells located at the vascular stalk of the glomerulus [1]. Podocytes are unable to undergo complete cellular division, and a loss of a critical number of podocytes is sufficient to trigger FSGS [2], [3]. PECs undergo cellular division throughout life [4] and are in direct continuity with podocytes at the vascular stalk. For this reason, it has been proposed that podocytes can be repopulated from PECs also in adult mammals [1], [5]. More recently, a crucial role of PECs has been shown in two major glomerular disease entities. Early cellular crescents in rapid progressive glomerulonephritis are formed exclusively by glomerular epithelial cells (PECs and podocytes) [6], [7]. Activated PECs have been observed also within the sclerotic lesions in patients affected by focal and segmental glomerulosclerosis (FSGS). Within these lesions, activated PECs deposit matrix changing the traditional concept about this disease [8], [9]. These findings have been made possible by the development of transgenic tools and mouse lines, that allow the specific manipulation of PECs *in vivo* [1]. Genetic tagging and lineage tracing are powerful tools to study the behavior and physiology of a specific cell type *in vivo* or to prove the origin of specific cells in development or in a specific disease model. A major breakthrough for podocyte biology has been triggered by the generation of podocyte-specific transgenic tools [10]–[12] but the generation of podocyte cell lines, which recapitulate the phenotype *in vivo* as closely as possible (for review see [13], [14]) proved to be equally important.

An immortalized cell line derived from murine PECs has previously been described [15]. However, so far no reliable method to determine the origin of glomerular epithelial cells existed. In this study, specific genetic tagging was used to overcome this problem.

## Results

### Primary cultures of genetically tagged glomerular outgrowths

**Figure 1:**
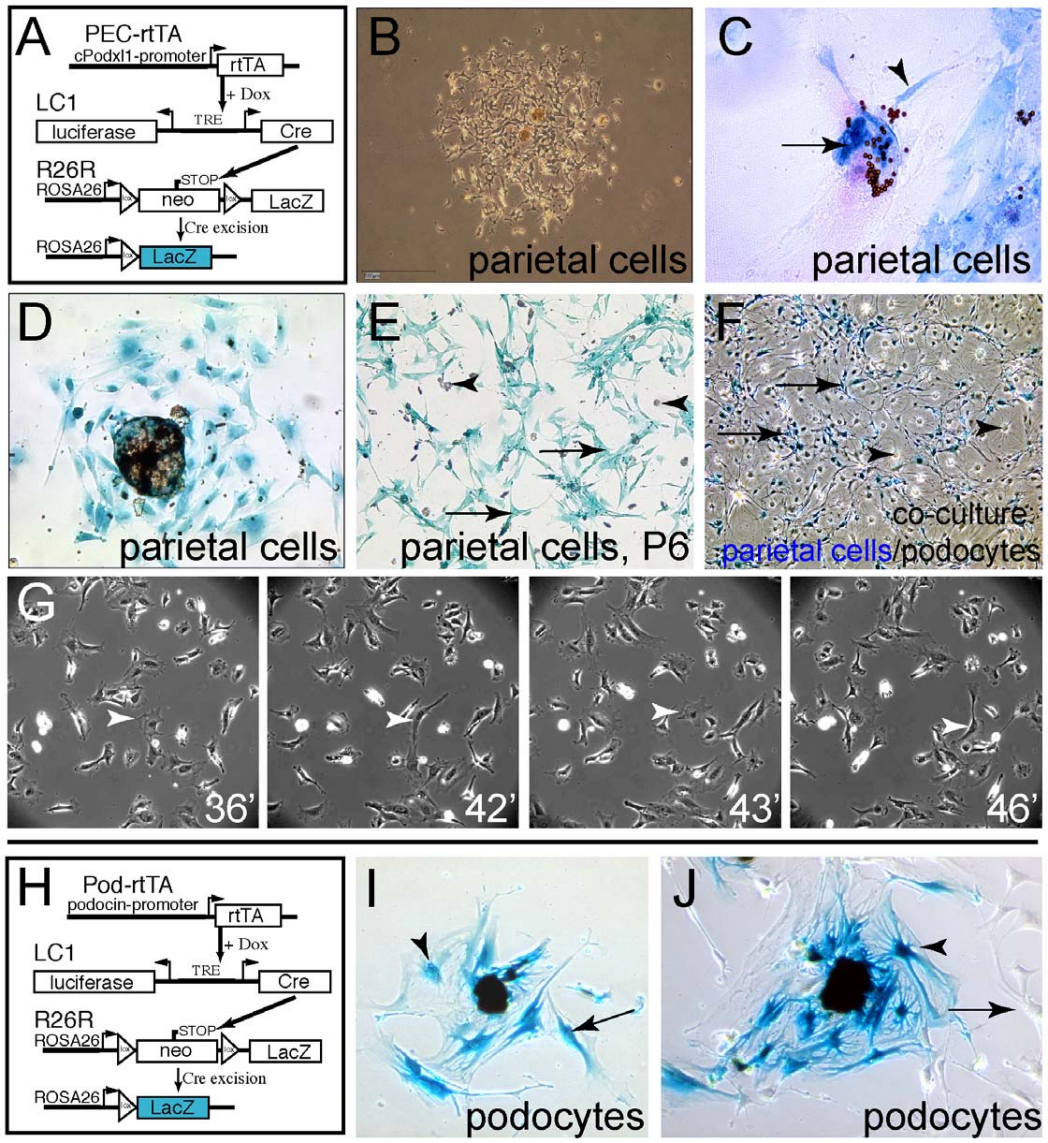
Cell lineage tracing of cellular outgrowths. A–E. Outgrowths from isolated capsulated glomeruli of PEC-rtTA/LC1/R26R mice are predominantly derived from genetically tagged parietal cells. A. Genetic map of triple transgenic PEC-rtTA/LC1/R26R mice (cPodxl, rabbit podocalyxin promoter; rtTA, reverse tetracycline transactivator; Dox, doxycycline). B. Phase contrast image of cellular outgrowths from two capsulated glomeruli. C. Cellular outgrowths are derived from parietal cells as shown by X-gal staining (arrowhead). Within the glomerulus, magnetic beads used for isolation are still visible. D. Genetically tagged parietal cells at later time points (X-Gal staining, after 7 days of culture). E. After six passages, genetically tagged parietal cells show a spindle-shaped morphology (arrows; X-Gal crystals, arrowheads). F. A mixed culture of untagged primary podocytes and genetically tagged primary parietal cells was established to compare the morphology of the two cell types. Beta-gal-negative podocytes formed mostly larger cells (arrowheads) compared to beta-gal-positive parietal cells, which were mostly spindle-shaped (arrows, X-gal staining). G. Stills from time-lapse video (Video S1, seconds indicated on lower right, 1 sec. = 75 min. capture time) of pure cultured parietal cells (see below). Arrowhead indicates a cell transitioning between spindle-shaped and polygonal morphology. H–J. Cellular outgrowths from decapsulated glomeruli of genetically tagged Pod-rtTA/LC1/R26R mice (after 7 or 10 days). H. Genetic map of the transgenic mice. I–J. In most cases, primary podocytes (blue) show a distinct morphology (larger cell body with prominent cytoplasm, arrowhead). For comparison, other cells without a genetic tag can be seen (J, arrow).

In order to trace the origin of cellular outgrowths, parietal cells were specifically and irreversibly labeled by administration of doxycycline (Dox) in female triple transgenic PEC-rtTA/LC1/R26R mice (Fig. 1A). In previous studies, we have shown that approximately 70% of parietal cells undergo Cre recombination and activate constitutive expression of beta-galactosidase in this mouse model. No other glomerular cells are labeled, specifically no podocytes [1], [7].

After a washout of at least 7 days, capsulated glomeruli were isolated and subjected to culture. Cellular outgrowths emerged after 4–7 or after 7–10 days using EGM-MV or RMPI media, respectively. Most of the cells were of a spindle-shaped or squamous morphology with multiple lamellipodia and a high proliferative activity (Fig. 1B). As shown by X-gal stainings, the majority of cells emerging from the capsulated glomeruli were genetically labeled parietal cells (Fig. 1C, D). Some beta-gal-negative cells showed a similar morphology to beta-gal positive PECs, most likely representing unlabeled parietal cells. Primary parietal cells conserved their morphology at least during the first six passages of culture in EGM-MV (Fig. 1E, passage 6, P6). When co-culturing genetically tagged parietal cells with outgrowths from decapsulated glomeruli (i.e. presumptive podocytes), the two cell types showed a different and characteristic morphology in the majority of cases (Fig. 1F). Genetically tagged primary PECs predominantly showed a spindle-shaped or squamous (polygonal) morphology (similar to PECs *in vivo*) while untagged presumptive primary podocytes were mostly larger in size and formed multiple intracytoplasmic extensions radiating from the nucleus into the periphery (Fig. 1F, arrowheads). As shown in Video S1 and representative stills (Fig. 1G), parietal cells transition between the spindle-shaped and polygonal phenotype in culture.

### Morphology of primary podocytes

In order to analyze primary podocytes in more detail, primary cellular outgrowths were generated from triple transgenic Pod-rtTA/LC1/R26R mice. In these mice, more than 70% of the podocytes had been specifically labeled by transient Dox administration as described above (Fig. 1H) [7]. Using X-gal stainings on primary outgrowths, it was verified that more than 50% of the primary podocytes exhibited the morphology described above (large cells with multiple intracytoplasmic extensions, i.e. thickenings, Fig. 1I, J). In the early primary outgrowths, the remaining podocytes also showed a more spindle-shaped morphology and smaller size, similar to parietal cells (Fig. 1I, arrow).

### Derivation of primary cultures from podocytes or parietal cells

**Figure 2:**
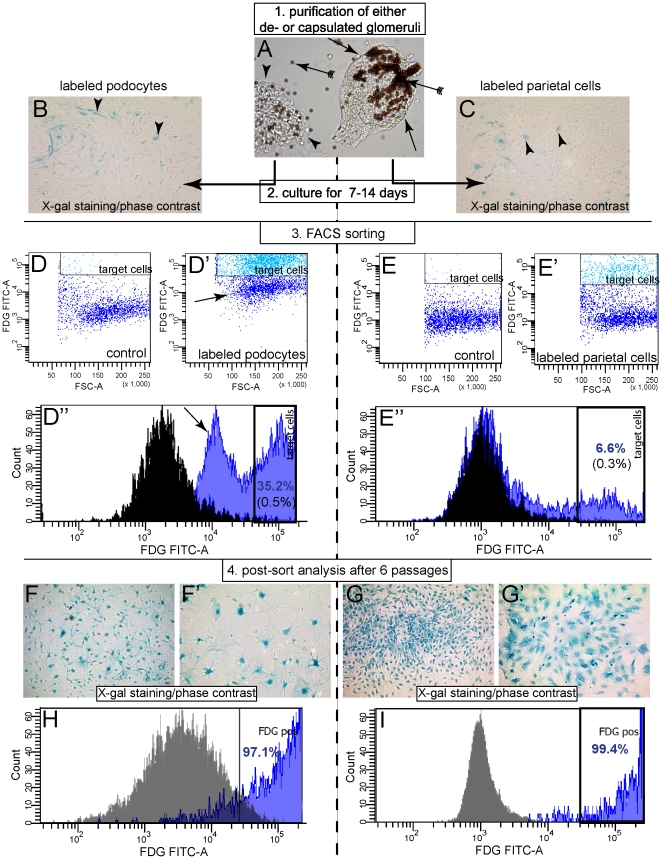
Generation of primary cultures of murine podocytes or parietal cells. A. First capsulated or decapsulated glomeruli were purified using magnetic beads (arrow with tails, >90% purity), to prepare primary mixed podocyte or parietal cell cultures respectively. For direct comparison, a capsulated glomerulus (arrows) is shown next to a decapsulated glomerulus (arrowheads). B, C. After 7–14 days of culture in EGM-MV media, primary outgrowths were obtained and stained for the genetic marker (X-gal). D, E. Next, suspensions of primary cells were treated with a luminescent substrate for beta-galactosidase (“labeled”, D′, E′) or not (controls, D, E) and were FACS-sorted using the indicated gate (“target cells”). In the example shown, 35,2% of the cells were defined as podocytes (D″) and 6.6% of the cells were defined as parietal cells (E″). F, G. The purity of enriched primary podocytes (F, F′) and parietal cells (G, G′) was evaluated after six passages in culture. Staining for the genetic marker beta-gal (X-gal) revealed that virtually all of the cells were either derived from podocytes or parietal cells (merged images: phase contrast+X-gal stainings). H, I. Similarly, in a FACscan analysis, virtually all cells were positive for the genetic tag beta-galactosidase (as revealed by metabolic labeling with FDG). In this representative analysis, 97.1% and 99.4% of the primary podocytes or PECs were defined as beta-gal positive, respectively.

Specific genetic labeling allowed, for the first time, the generation of primary parietal cells or podocytes using FACS cell sorting (Fig. 2). In brief, either podocytes or parietal cells were genetically labeled in triple transgenic mice by administration of Dox for 14 days as described above (see Figs. 1H and A, respectively). After a washout period of 7 days, de-capsulated or capsulated glomeruli were prepared from female Pod-rtTA/LC1/R26R or PEC-rtTA/LC1/R26R mice, respectively. Glomerular preparations were >90% pure, as verified by phase contrast microscopy (Fig. 2A). Glomerular preparations were cultured for 7–14 days to obtain primary cellular outgrowths. About 50±25% or 20%±15% of primary outgrowths were derived from the podocyte or parietal cell lineage, respectively. This was verified by enzymatic staining for the genetic marker beta-galactosidase (Fig. 2B,C). Labeling frequency was lower in parietal cells, because of the more prominent mosaicism in the parietal mouse (PEC-rtTA [1], [7]). Single-cell suspensions of primary cultures were treated with fluorescein di-beta-D galactopyranoside (FDG), which is hydrolyzed by beta-galactosidase (beta-gal) into a fluorescent product, and subjected to FACS sorting. The target cells could be identified as a specific distinct cell population (Fig. 2D–D″, E–E″ “target cells”). In podocyte preparations, increased background fluorescence was noted in the negative cell population if the cells were suspended in HBSS instead of RPMI (Fig. 2D′ and D″, arrows). Nevertheless, both populations were still clearly distinct from each other even when using HBSS. After FACS sorting, the cells remained viable. In general, about 2–5×10^5^ cells derived from podocytes and 2–5×10^4^ cells derived from parietal cells were obtained per adult mouse (as verified in a post-sort FACS analysis). After six passages in culture, we could not find any evidence for non-labeled contaminating cells. Of note, when culturing primary podocytes in EGM-MV media at low cellular densities, their characteristic morphology (large cells with intra-cytoplasmic extensions) remained preserved even after more than six (Fig. 2F–G′) or nine passages (not shown). Similarly, the vast majority of cells were beta-gal positive as shown by metabolic labeling with FDG and subsequent FACS analysis (Fig. 2H, I, gray, negative control without FDG). After 9 passages in culture, primary cultures were still virtually free of non-labeled cells (not shown).

### Transcriptome analysis of primary podocyte and parietal cell cultures

**Figure 3:**
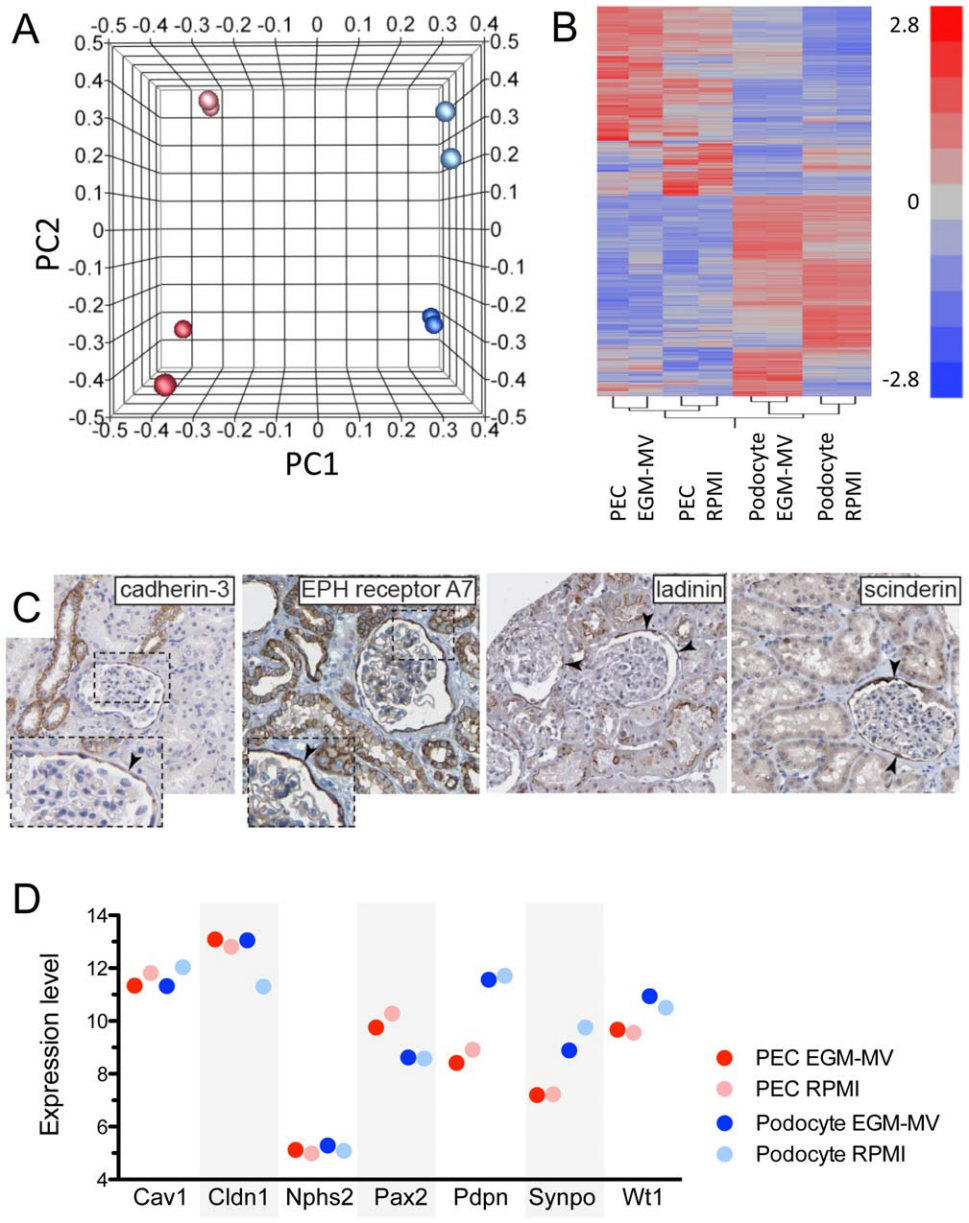
Transcriptome analysis of primary podocyte and PEC cultures. A. Eight transcriptomes were determined from two independent primary podocyte cultures and two independent primary PEC cultures grown in two different media (EGM-MV+20% FCS or RPMI+10% FCS), respectively. Transcriptomes were subjected to principal component analysis. PEC cultures (red spheres) are separated from podocyte cultures (blue spheres) along the axis of principle component 1 (PC1), which accounts for 47.8% of the overall variance in gene expression. Cells cultured in RPMI (light red and light blue spheres) segregate from those cultured in EGM-MV (bright red and bright blue spheres) along the axis of PC2, accounting for 11.8% of the variance. B. Primary podocyte and PEC cultures can also be clearly distinguished by cluster analysis of 2,507 significantly differentially regulated genes. C. Differential expression of the gene mRNA transcripts cadherin-3, EPH receptor A7, ladinin and scinderin in primary PEC cultures correlated with preferential expression in PECs versus podocytes in human kidney *in vivo* (arrowheads; images from www.proteinatlas.org [16]). D. Expression levels of selected genes in primary podocyte and PEC cultures. Expression levels are given as mean logarithmic values to the base 2 of arbitrary intensity units.

Two independent primary podocyte and parietal cell preparations were characterized on a genome-wide scale after six passages. To this end, the transcriptomes were determined by microarray analysis (see File S1 for primary data). To investigate the influence of different media, both cell preparations were cultured either in RPMI+10% FCS or EGM-MV+20% FCS. Thus, eight transcriptomes were determined in total. Principle component analysis (PCA) was used to identify the major source of variation in gene expression between the eight transcriptomes (Fig. 3A). The largest variance (47.8%) was explained by the difference in gene expression between the two cell types (principle component 1, PC1, between podocytes versus PECs). On the other hand, the two culture media accounted only for 11.8% of the total variance (PC2). These results indicated that primary cultures of PECs and podocytes retained significant differences in gene expression depending on their origin. The type of culture medium contributed only a minor influence to the similarity or dissimilarity between primary podocyte and PEC cultures - although the media influenced significantly the morphology of primary podocytes as shown above.

Overall, expression of 2,507 genes out of the 22,237 monitored genes differed significantly among the eight transcriptomes. Cluster analysis of the significantly differentially expressed genes confirmed the segregation of primary cultures with respect to the origin of the cells (podocyte vs. PEC) and – to a lesser extend – to culture media (RPMI+10% FCS vs. EGM-MV+20% FCS) (Fig. 3B).

As shown in File S1, the transcriptomes were analyzed to identify genes that were differentially regulated between primary podocytes and PECs and which were not significantly influenced by the media EGM-MV and RPMI (i.e. by less than a factor of 2). Primary podocytes expressed relatively high mRNA levels of biglycan, RhoGTPase activating protein 28, podoplanin, integrin beta V, synaptopodin and LIM homeobox transcription factor 1 beta. *In vivo*, many of these transcripts are expressed in a podocyte-specific fashion (for reference see www.proteinatlas.org [16]). Similarly, when evaluating the expression pattern of PEC-specific transcripts, several genes were identified which were also expressed by PECs *in vivo*, while podocytes were negative (i.e. cadherin-3, EPH receptor A7, ladinin and scinderin, www.proteinatlas.org, Fig. 3C). This indicated that some characteristic protein expression patterns remain preserved in primary PEC cultures. Next, expression levels of selected genes were analyzed, which have been implicated in discerning primary podocyte and PEC cultures (Fig. 3D). Podocyte as well as PEC cultures expressed caveolin-1, claudin-1, Pax2, but not podocin (Nphs2) or nephrin (Nphs1 with a log(2) expression value of ≈5.5). The expression levels of these genes were somewhat similar between podocyte and PEC cultures. A differential expression was observed in primary podocyte cultures versus primary PECs for synaptopodin and podoplanin, and also for WT-1 although to a lesser extent (Fig. 3D).

### Characterization of primary cell cultures

**Figure 4:**
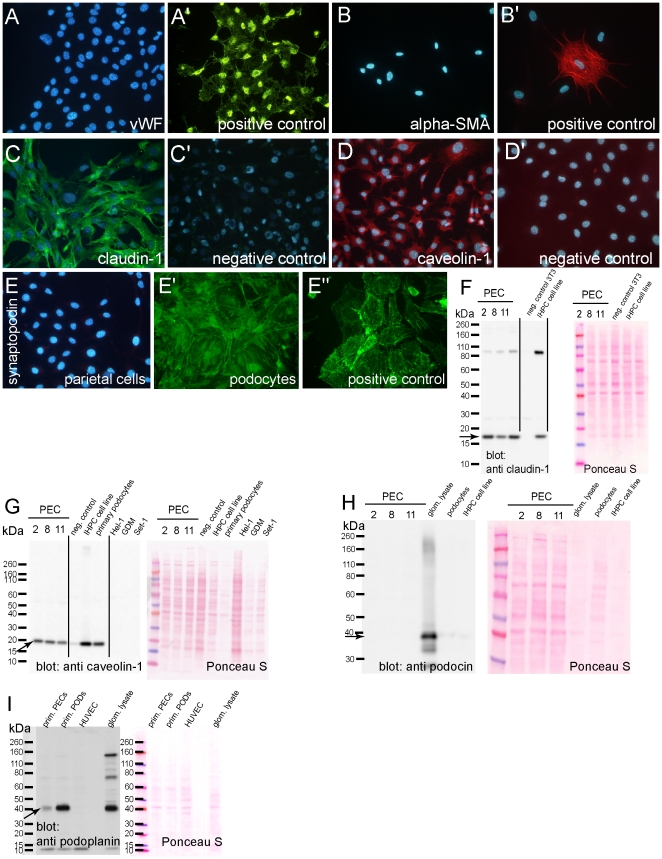
Characterization of the primary PEC lines. PECs were negative for the endothelial marker *von Willebrand factor* (vWT, A) and for myofibroblast marker *alpha-SMA* (B). HUVECs (A′) or human dermal fibroblasts (B′) were used as positive controls. Polyclonal parietal cells were positive for claudin-1 (C) and caveolin-1 (D). C′, D′. Negative controls were performed using isotype-matched irrelevant primary antibodies. E. A significantly lower expression of synaptopodin was observed in parietal cells expressed even after six passages compared to primary podocytes (E′) or an immortalized podocyte cell line IHPC (E″). The findings were confirmed by SDS-page with subsequent immunoblotting using lysates of parietal cell cultures 2, 8 and 11 (F–H). Lysates of polyclonal primary podocyte cultures as well as of an immortalized podocyte cell line IHPC also showed expression of claudin-1 and calveolin-1. No expression of caveolin-1 was observed in Hel-1, GDM or Set-1 cell lines (G). Equal loading was verified by Ponceau S stain. H. Podocin expression is absent in primary PECs and down regulated in primary podocyte cells and in an immortalized podocyte cell line IHPC. I. Podoplanin is differentially expressed in primary podocytes relative to primary PECs after six passages in culture (arrow), consistent with mRNA expression analysis. Lysates of isolated glomeruli are used as positive control (H, I).

Primary cell cultures were characterized in more detail by immunohistology and immunoblotting. Most importantly, it was verified that primary parietal cells did not express endothelial or mesangial cell markers (von Willebrandt factor, vWF; Fig. 4A, A′ or alpha-SMA; Fig. 4B, B′, respectively). As already indicated by transciptome analysis, the parietal marker claudin-1 was expressed by PECs (Fig. 4C, C′) and confirmed by immunoblotting (Fig. 4F). Claudin-1 expression was also noticed in an immortalized podocyte cell line IHPC when grown under non-permissive conditions (38.5°C, no INF-gamma) and also in primary podocytes (not shown). Caveolin-1, a second parietal cell marker, was also expressed by primary parietal cells as detected by immunofluorescence (Fig. 4D, D′) or immunoblotting (Fig. 4G). Expression of caveolin-1 was also detected in lysates of primary podocytes as well as of IHP cells under non-permissive conditions. Similar to our results of the transcriptome analysis, expression of synaptopodin was significantly higher in podocytes compared to PECs after six passages in culture (Fig. 4E, E′). No *de novo* expression of podocin was detected by immunoblotting in PECs (Fig. 4H) and podocin expression was down regulated after six passages of culture in primary podocytes and in IHPC cells (again consistent with our transcriptome analysis). Finally, differential expression of podoplanin was verified by immunoblotting lysates of primary PECs and podocytes after six passages in culture. Consistent with our transcriptome analysis, podoplanin was differentially expressed in primary podocytes compared to PECs.

### WT-1 and Pax2

**Figure 5:**
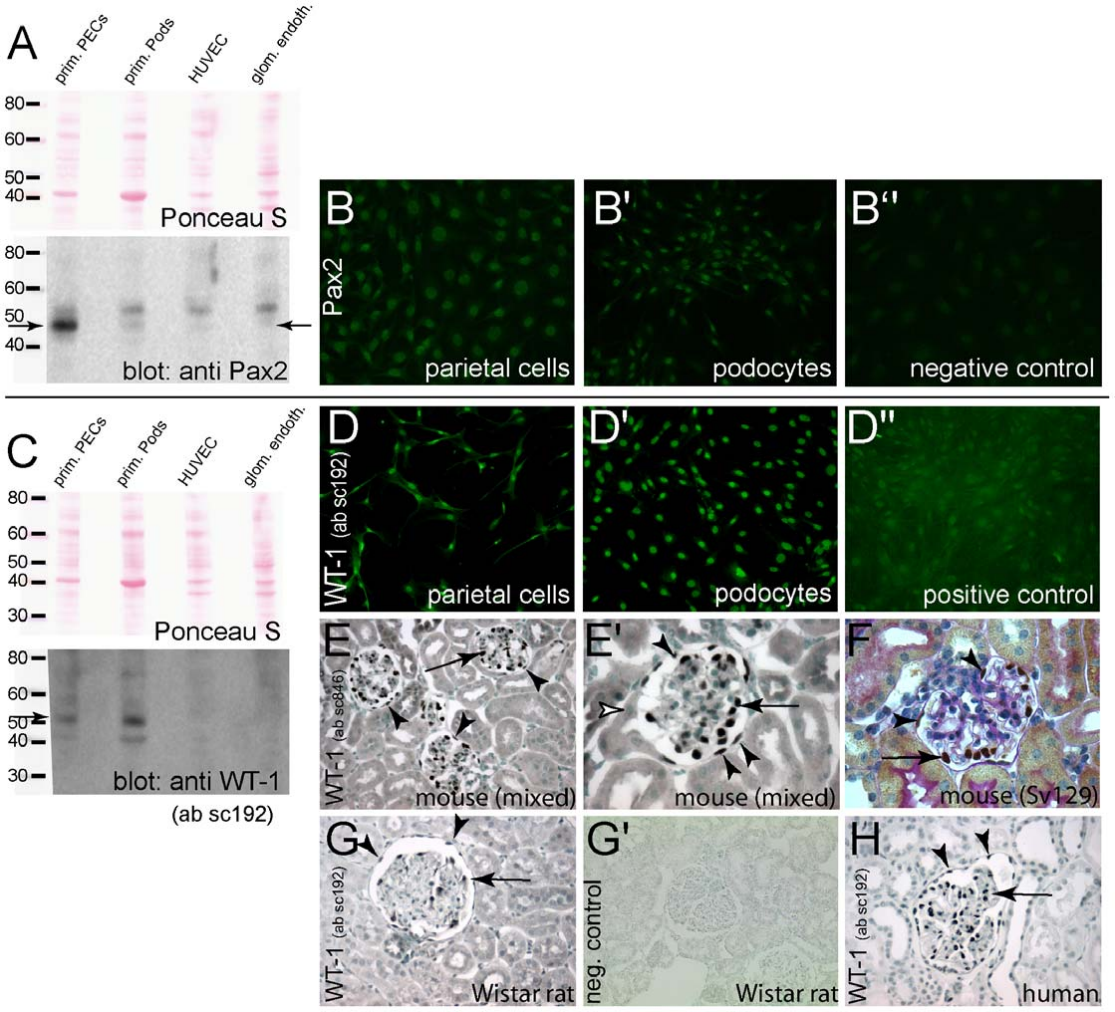
Expression of podocyte marker proteins by primary parietal or podocyte cell lines. A. Lysates of primary PECs or podocytes were subjected to immunoblotting for Pax2 expression. Primary PECs expressed significant amounts of Pax2, lower amounts were detected in primary podocytes (arrow). Lysates of endothelial cells (HUVEC or glomerular endothelial cells) were used as negative controls. B. After six passages, Pax2 was expressed in primary PECs and podocytes (arrow). Total kidney lysates were used as positive controls, endothelial cells were used as negative controls. C, C′ In immunofluorescent stainings on primary PECs and podocytes after six passages of culture, Pax2 was expressed in a nuclear fashion. Isotype-matched irrelevant antiserum was used as control (C′). D. Immunoblotting lysates of primary PECs or podocytes using ab sc192 showed that WT-1 is expressed in both cell types with significantly higher levels in primary podocytes (arrow). Lysates of endothelial cells were negative for WT1. E. After six passages, WT1 was expressed both in PECs and podocytes in a nuclear fashion (immunofluorescent staining using ab sc192). F. Parietal cells express low levels of WT1 also *in vivo* in mice with a mixed genetic background using ab sc846 (F, F′, arrowheads, immunohistological staining). For comparison, a strong expression of WT-1 was detected in podocytes (arrows). No WT-1 expression was noted elsewhere in the renal cortex, specifically not in proximal tubular cells (white arrowheads). G. Similarly, PECs expressed WT-1 also in other mouse genetic backgrounds (Sv129; WT-1/PAS staining) H, I. PECs (arrowheads) expressed low amounts of WT-1 also in normal Wistar rats (H) and humans (I). H′. Isotype matched controls were negative.

Transcription factors WT-1 and Pax2 are considered to play a crucial role in renal development and are differentially expressed in PECs and podocytes [17]. So far, WT-1 has generally been used as a podocyte marker. To determine the expression pattern of these two transcription factors, lysates from primary PECs or podocytes were prepared after FACS sorting and subjected to SDS-page and immunoblotting. As shown in Fig. 5A, Pax2 was strongly expressed in primary PECs (arrow) – similar to PECs *in vivo*. Interestingly, a weaker band was also detected in primary podocytes. Endothelial cells (HUVEC) were used as negative controls. After six passages, Pax2 was expressed in primary PECs but also in primary podocytes (Fig. 5B). Persistent nuclear Pax2 expression was also confirmed by immunofluorescence (Fig. 5C). The transcription factor WT-1 was expressed already in primary podocytes, as shown by immunoblotting (Fig. 5D). Notably, WT-1 was also expressed in primary PECs (Fig. 5D). Lysates of HUVEC were used as negative controls. After six passages, primary PECs and podocytes still expressed significant levels of WT-1 within the nuclei (Fig. 5E). An immortalized podocyte cell line, IHPC, was used as positive control (Fig. 5E″). These results were nicely corroborated by our transcriptome analysis of Pax2 and WT-1 (Fig. 3C). To test if WT-1 is also expressed in parietal cells *in vivo*, immunohistological stainings from normal mouse kidneys were performed using two different antibodies (ab) and renal tissues of different species (Fig. 5F–I). As described previously, a significant nuclear staining within the podocytes was noticed (arrows). However, the nuclei of parietal cells were also positive for WT-1 – albeit to a lesser extent (arrowheads). No WT-1 expression was observed within proximal tubular cells lining Bowman's capsule (white arrow). Similar results were obtained in renal tissues from mice with a mixed (F, F′) or Sv129 genetic background (G), from Wistar rats or humans (H–I). It was concluded, that WT-1 is also expressed by parietal cells *in vivo*.

### Analysis of primary cellular outgrowths from decapsulated or capsulated glomeruli

**Figure 6:**
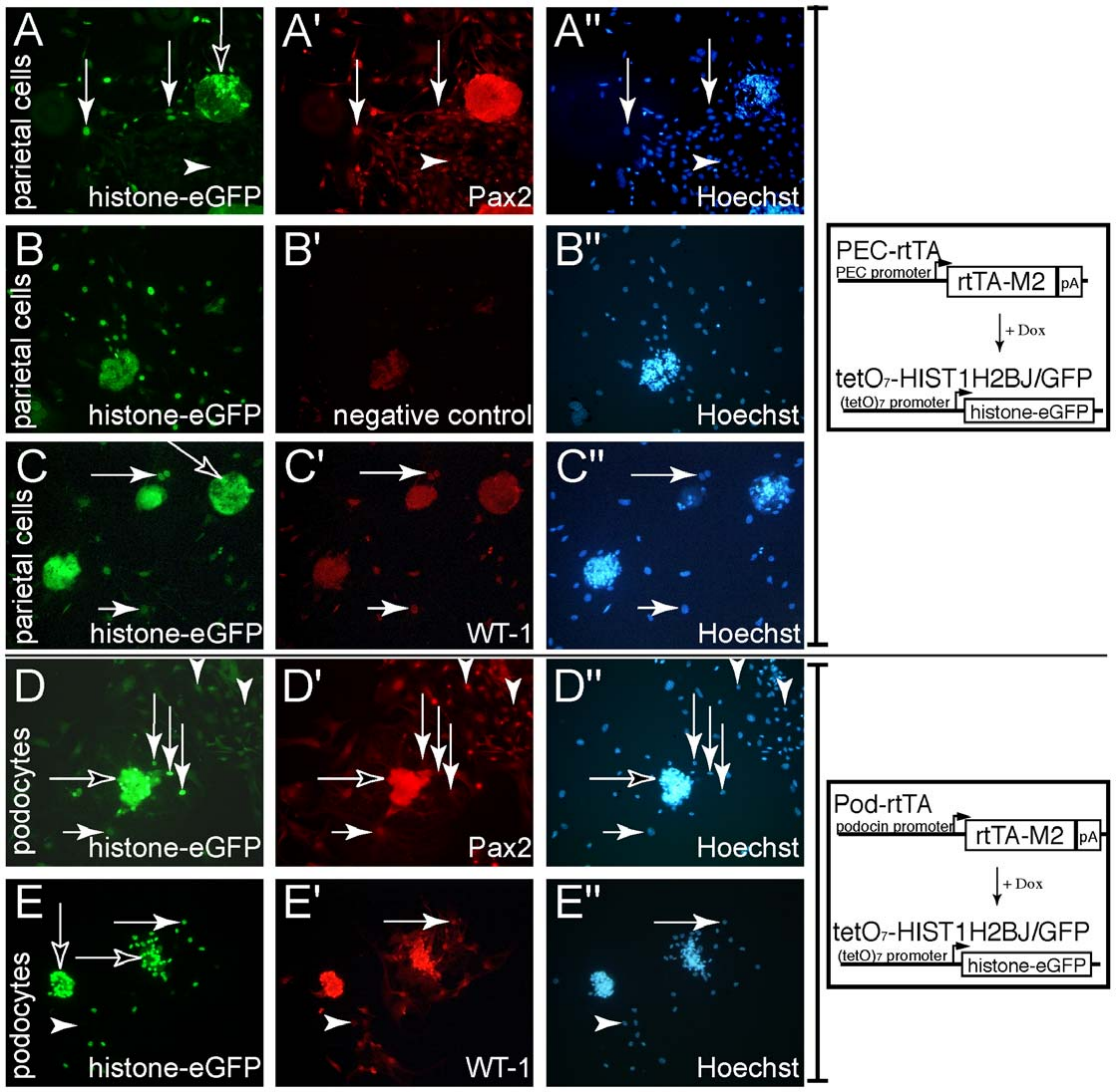
Expression of Pax2 and WT-1 in cellular outgrowths of primary parietal cells and podocytes. Parietal cells were fluorescently labeled in transgenic PEC-rtTA/(tetO)-HIST1H2BJ/GFP mice (A–C), which express histone-eGFP in a doxycycline-inducible fashion in parietal cells but not in podocytes. After labeling of parietal cell nuclei was completed in living mice (genotypes are indicated on the right), the glomeruli were isolated and the cellular outgrowths were analyzed. A–A″, eGFP labeled nuclei can be observed within the capsule of a glomerulus (black arrow). Labeled cellular outgrowths can be observed (white arrows). Unlabeled cellular outgrowths – most likely podocytes - originate from a decapsulated glomerulus (arrowhead). Pax2 is expressed within the nucleus by the majority of cellular outgrowths independent of their origin from parietal cells or podocytes (A′). B, Control staining from the same experiment as in A using irrelevant primary antiserum. Background staining can be observed within the three-dimensional structures of the glomeruli and outside the nucleus of some cells. C, Cellular outgrowths from capsulated glomeruli (black arrow) express WT-1. Nuclear expression of WT-1 can be detected in all cellular outgrowths derived from GFP positive parietal cells (arrows). D, Cellular outgrowths from decapsulated glomeruli of Pod-rtTA/Hist1H2BJ/GFP transgenic mice (black arrows) upregulate Pax2 expression. Pax2 is not expressed within the nuclei of early podocyte outgrowths (white arrows with long tails). Pax2 expression is upregulated in podocyte-derived outgrowths with lower nuclear histone-eGFP levels, presumptively because these cells have already undergone cellular divisions (white arrow with short tail). Cellular outgrowths without histone-eGFP labeling (proliferating podocytes or parietal cells) express the highest levels of Pax2 (arrowheads). E, EGFP labeled podocyte-derived cells express WT-1 in a nuclear pattern (white arrow). EGFP negative (and presumably less differentiated) cells express significantly less WT-1 (white arrowhead).

In order to analyze the differentiation status of very early primary outgrowths from isolated glomeruli in more detail, parietal cells (PECs) or podocytes were specifically labeled by transient expression of histone-eGFP (Fig. 6). Upon administration of Dox using a bigenic system (PEC- or Pod-rtTA/tetO_7_-Hist1H2BJ/GFP), the nuclei of parietal cells were loaded with histone-eGFP, a protein with a very long half-life [18] (Fig. 6A). With this labeling, double fluorescent stainings could be performed. In addition, the intensity of the eGFP labeling correlates inversely with the number of cellular divisions, since no additional expression of histone-eGFP occurred in the absence of Dox. Nuclear eGFP labeling is lost entirely in proliferating cells after more than six cellular divisions (own observations).

Early cellular outgrowths were analyzed in primary PECs using the histone-eGFP labeling method. A generalized expression of Pax2 was observed in early cellular outgrowths (for a representative example see Fig. 6A′). Pax2 expression was higher in eGFP-labeled primary PECs (positive correlation between eGFP and Pax2 fluorescent intensities). WT-1 was also expressed at lower levels in primary PECs in culture (Fig. 6C).

When analyzing Pax2 expression in primary podocytes, a de-novo expression was observed in early cellular outgrowths (Fig. 6D). Primary podocytes with high eGFP intensities and located close to the decapsulated glomerulus did not express detectable levels of Pax2 (Fig. 6D, long white arrows). However, a weak de novo expression of Pax2 could be observed in podocytes with weak eGFP labeling (Fig. 6D, short white arrows). WT-1 expression on the other hand was down regulated in primary podocytes but WT-1 expression persisted in eGFP low or negative cells (Fig. 6E).

## Discussion

In the present work, we describe four major findings. First, we have used the technology of irreversible genetic tagging to unequivocally determine the origin of cellular outgrowths from isolated glomeruli in culture. With this technology, we could overcome a long known fundamental issue that almost all cells undergo significant changes of their phenotype in culture so that their origin can no longer be determined with certainty. Even with our improved protocol, it is almost impossible to enrich for purely decapsulated glomeruli using the conventional techniques available. It has been shown, that even a fraction of only 5–10% of capsulated glomeruli are sufficient to obtain primary cultures of cells that originated most likely from parietal cells [3]. Furthermore, it was well documented that remnant parietal cells located at the vascular stalk emerge even from decapsulated glomeruli [3], [19].

In this study, the primary cultures retained a distinct and highly reproducible expression profile depending on their origin even after six passages in culture. It was previously shown, that podocytes loose expression of some specific markers, such as podocin or nephrin [14], as also confirmed in our study. Interestingly, the influence of different media (either RPMI or the growth-factor enriched EGM-MV) had only a minor influence on the transcriptional profile. Of note, several gene transcripts with differential expression between primary PEC and podocyte cultures could be identified in this study, which are also differentially expressed *in vivo*. Two of these might even be useful as a marker for primary podocytes in culture (i.e. synaptopodin and podoplanin). These results are very reassuring that primary cultures with defined origin have the potential to be a useful model to the field of glomerular biology.

Second, we could resolve the morphology of primary cells originating from podocytes or parietal cells. Primary parietal cells were spindle-shaped or squamous (polygonal, flat) cells forming multiple lamellipoda. Podocytes showed two different morphologies: On the one hand, they formed large flat cells with intracytoplasmic thickenings that extended in a radial fashion from the nucleus into the periphery, so that the cells appeared “arborized”. This morphology was characteristic for primary podocytes, it was never observed in parietal cells and thus it can be used to identify the origin of podocytes in culture – albeit only as a positive marker since podocytes could also form a phenotype similar to parietal cells, as described above. In time-lapse videos, we could observe that the primary outgrowing podocytes may switch from a PEC-like morphology to a Pod-like morphology and vice versa (not shown). Mundel *et al.* has already shown that podocytes can be pushed towards the arborized Pod-like phenotype by specific culture conditions [20]. In this study, we could select for the characteristic morphology of cultured podocytes using EGM-MV medium +20% FCS in combination with low cell densities.

Our findings are consistent with the observations of other investigators [3], [19], [21]–[23], who have already observed the distinct cellular morphologies. Interestingly, these investigators have also observed both morphologies emerging from glomeruli independent of the presence of a capsule with PECs. Our study clarifies, that primary podocytes may show different phenotypes and thus provides an explanation for the results of the previous studies.

Third, our result allow to conclude that podocytes can undergo repeated cellular divisions *in vitro*. Even after more than 35 passages, primary podocytes with proven origin proliferated in culture. Primary parietal cells could also be kept in culture for more than 35 passages and no obvious phenotypic changes were observed after prolonged culture (not shown). We were able to prepare individual clones of parietal cells by limited dilution (not shown). In primary podocytes, subcloning using limited dilution was not possible with these cells (not shown). Prolonged culture was only performed as additional information on the proliferative behavior of these cells. The characterizion of the cells was focussed on the early passages (i.e. passage 6), which are relevant for biological experiments. Podocytes are generally believed to be terminally differentiated, post-mitotic cells, so that they are unable to undergo complete cellular divisions under normal conditions [24], [25]. Some groups have observed that podocytes display only a very limited mitotic capacity when subjected to culture [5], but so far it has been impossible to trace podocytes in culture.

We and others have shown that podocytes participate in the formation of cellular crescents in rapidly progressive glomerulonephritis [6], [7], [26], [27]. Cellular crescents are extracapillary proliferations derived from podocytes and – potentially even more important - from parietal cells. They contribute to loss of renal function by blocking the tubular outflow of the primary urine [28]. In this context, our finding that podocytes can undergo repeated cellular divisions in culture supports our previous findings in proliferative glomerular diseases.

Forth, we show clearly that WT-1 is expressed by parietal epithelial cells (PECs) *in vivo* as well as *in vitro*. When PECs are subjected to culture, all PECs express WT-1 even shortly after emerging from the glomerulus. In primary podocytes, WT-1 was expressed at significantly higher levels but over time WT-1 was down regulated to expression levels comparable to that of PECs – even in podocytes with an arborized phenotype. Therefore, it was concluded that WT-1 cannot be used as a podocyte-specific marker gene.

We also observed that Pax2 is expressed de novo in primary podocytes. After prolonged culture, podocytes as well as PECs expressed similar levels of Pax2 similar to WT-1. In our hands, the markers that were expressed at higher levels in podocytes were synaptopodin and podoplanin. From these results, we conclude that parietal cells and podocytes cannot be unambiguously distinguished in culture using conventional markers.

Finally, this work establishes for the first time primary parietal cell and podocyte cultures with proven origin using a reliable and reproducible methodology. In addition to using genetic tagging of the primary target cells, several additional methodological improvements were implemented (e.g. an improved protocol to isolate capsulated glomeruli).

Although changes of the phenotype occur in all cells in culture, primary cell culture probably still mimics closest the situation *in vivo*. FACS sorting of genetically labeled cells allowed the preparation of primary cultures. These primary cellular preparations could be frozen and revitalized, so that these primary cells are a significant novel contribution to the *in vitro* models of glomerular research and have the potential to be a useful novel experimental model for glomerular research.

## Materials and Methods

### Animals

All transgenic animals were housed under SPF-free conditions. All procedures were approved by the German government officials (LANUV NRW 8.87–51.05.20.09.251; 8.87–51.05.20.11.038, total number of animals = 32). To induce the genetic labeling, animals received doxycycline hydrochloride (Fargon GmbH&Co, Barsbüttel, Germany) via drinking water for 14 days followed by at least 7 days of washout as described [1].

### Perfusion and isolating glomeruli

Mice were anesthetized using ketamin/rompun and perfused via the left ventricle with magnetic beads (Dynabeads M450 Tosylactivated Lot: 472610 Invitrogen Oslo, Norway) diluted in 20 ml 0,9% NaCl. Kidneys were transferred into RPMI 1640 medium (Invitrogen) containing 1% penicillin/streptomycin and cut into small fragments. To enrich for decapsulated glomeruli these fragments were treated for 30 minutes at 37°C with 1 mg/ml collagenase TypIV (49H11312, Worthington). This step was omitted to enrich for capsulated glomeruli. The small kidney fragments were gently sieved through a 100 µm strainer, centrifuged and resuspended in PBS. The glomeruli were isolated using a magnetic particle concentrator (DynaMag TM-2, 123.21D, Invitrogen).

### FACS sorting

Capsulated or decapsulated glomeruli were isolated from transgenic Pod-rtTA/LC1/R26R or PEC-rtTA/LC1/R26R mice, respectively, and cultivated for 7–14 days in EGM-MV media. After trypsinization, the cells were suspended in RPMI 1640 medium (21875 Invitrogen) +1% pen/strep (to reduce fluorescence background) and treated for 1 minute at 37°C with 2 mM fluorescein di-ß-galactopyranoside (FDG, F1179, Molecular Probes). Subsequently the samples were diluted 10-fold with RPMI media for further incubation on ice for 60 minutes. The emission at 514 nm was detected using a BD FACSAria II cell sorter. The sorted cells were cultured as described.

### Cell culture

Primary parietal cells and podocytes were cultured in RPMI+10% fetal calf serum (FCS) (Biowest Florida, United States) or EGM-MV (Lonza Basel, Switzerland) +20% fetal calf serum as indicated. EGM-MV is enriched for growth factors and FCS to promote survival of primary cells in culture, it was used to isolate human parietal cells by Ronconi *et al.* [1], [5]. Cells were passaged when 70–90% confluency was reached and were seeded at 1–5×10^5^ cells/ml media. Primary cells should be propagated in EGM-MV during the first three passages. Primary PECs can subsequently be cultured in RPMI. HUVEC's (human umbilical vein endothelial cells and ihGEC (immortalized human glomerular endothelial cells) were cultured in EGM-2 medium (cc-4176 EGM-2 SingleQuot Kit Suppl. & Growth Factors, Lonza). An immortalized human podocyte cell line IHPC [29] was cultured in VRAD Medium containing DMEM/F12 (Invitrogen) +10% fetal calf serum, 1% pen/strep, 1% L-glutamat (Invitrogen), all trans retinoic acid 1 µM and 25-hydroxycholecalciferol 1 nM (H4014, Sigma-Aldrich, St Louis, MO, USA). 3T3 Mouse and human dermal fibroblasts were cultured in RPMI 1640 medium +10% fetal calf serum, 1% pen/strep and 1% L-glutamine. COS7 cells (green monkey kidney cells) were cultured in DMEM medium (Gibco, Oslo, Norway) +10% FCS, 1% pen/strep and 1% L-glutamine.

### Beta-galactosidase assays

For enzymatic X-gal staining, the cells or glomeruli were fixed with 2% glutaraldehyde in PBS supplemented with 1 mM MgCl2 and 0,02% NP40. After three wash with PBS, the samples were incubated overnight at 28°C in a humidified atmosphere 1 mg/ml X-gal, 5 mM potassium ferricyanide, 5 mM potassium ferrocyanide, and 2 mM MgCl2 in PBS, pH 7.8 and mounted (Immu-Mount, Thermo Scientific, Waltham, MA). The images were analyzed using Leica DMR X microscope (Leica Microsystem GmbH Wetzlar, Germany) and collected with AnalySIS (Soft Imaging System, Muenster, Germany).

### Expression analysis

Two independent primary podocyte cultures and two independent primary PEC cultures maintained in EGMV+20% FCS or in RPMI+10% FCS were used for transcriptome analysis at passage number 6. Three days after the last medium change, RNA was isolated using Trizol (Invitrogen) followed by purification using the RNA Clean-Up and Concentration Micro Kit (Norgen). RNA quantity and purity was measured at 260/280/230 nm with a Nanodrop photospectrometer (Thermo Scientific). RNA integrity was assessed by using the Bioanalyzer 2100 (Agilent). Total RNA (200 ng) was reverse transcribed into cDNA, amplified, and *in vitro* transcribed to cRNA. Sense-strand cDNA was generated from 10 µg of purified cRNA using random primers, followed by fragmentation and labeling using 5.5 µg of purified sense-strand DNA. Biotinylated sense-strand DNA was then hybridized onto the Affymetrix GeneChip® Mouse Gene 1.0 ST arrays for 16 h. Arrays were washed and stained using the Fluidics Station 450. Scanning was performed by Scanner 3000 7 G (Affymetrix); raw CEL files were generated using the GCOS software. Data of the eight microarrays were analyzed with JMP Genomics 4.0 (SAS Institute Inc.) using the ENSG 14.0 custom CDF (Microarray Lab, Dept. of Psychiatry/Molecular and Behavioral Neuroscience Institute, University of Michigan, MI, USA). Data were log2 transformed, quantile-normalized and RMA background corrected. Data points with large residuals (outliers) were filtered out, using three iterations and a false discovery rate (FDR) of 0.05. Multiple testing was done using ANOVA and a post-hoc *t*-test with an FDR of 0.05, resulting in an adjustment of the p-value threshold. In our study, eight transcriptomes, each containing 22,237 genes, were compared. Thus, at an overall FDR of 0.05, individual p-values had to be lower than 0.00148 to be considered statistically significant. Transcriptomes were deposited at the Gene Expression Omnibus (GEO) database (Acc# GSE33714).

### Immunofluorescence

Cells were fixed with either −20°C acetone or 3% paraformaldehyde followed by permeabilization with 0,3% TritonX. Cells were incubated with primary antibodies and secondary antibodies diluted in 2% bovine serum albumin in PBS for 60 or 30 minutes, respectively. The following primary antibodies were used: polyclonal mouse anti-synaptopodin antibody (1∶100, 65294; Progen, Heidelberg, Germany), polyclonal rabbit anti–von Willebrand factor (1∶100, A0082; DAKO, Glostrup, Denmark), monoclonal mouse anti–alpha-smooth-muscle actin (1∶100, M0851, DAKO), polyclonal chicken anti-nestin antibody (1∶40, Abcam, Cambridge, UK), polyclonal rabbit anti-podocin antibody (1∶100, P0372 Sigma-Aldrich), polyclonal rabbit anti-caveolin1 antibody (1∶50, sc894, Santa Cruz Biotechnology, California, USA), monoclonal mouse anti-e-cadherin antibody (1∶800, BD610181, BD Bioscience, NJ, USA), monoclonal mouse anti-pan-cadherin antibody (1∶400, C1821, Sigma-Aldrich), polyclonal rabbit anti- claudin1 antibody (1∶50, ab15098-500, Abcam), polyclonal rabbit anti-pax2 antibody (1∶50, 716000, Invitrogen) polyclonal rabbit anti-wt1 antibody (1∶50, sc-192, Santa Cruz Biotechnology), monoclonal mouse anti-GFP (1∶100, 632381, Clontech), polyoclonal rabbit anti-GFP (1∶100, 632460, Clontech), rabbit or mouse serum (Dianova). Secondary antibodies used (Dianova): Cy2 rabbit anti-mouse (315-225-003 Lot. 58409), Cy3 goat anti-rabbit (111-165-003 Lot. 60256), Cy3 donkey anti-chicken (703-165-155 Lot. 71533), DyeLight549 donkey-anti mouse (1∶200, 715-505-151), DyeLight549 donkey anti-rabbit (1∶200, 711-505-152), DyeLight488 donkey anti mouse (1∶200, 715-485-151), DyeLight488 donkey anti rabbit (1∶200, 715-505-152). Nuclear staining HOECHST 33342 (Sigma Aldrich). The stained cells on glass coverslips were mounted onto a glass slide with Immumount (Thermo ScientificImmu-Mount). The stainings were analysed using Leica DMR X microscope (Leica Microsystem GmbH Wetzlar, Germany) and collected with AnalySIS (Soft Imaging System, Muenster, Germany).

### Immunoblotting

Cellular lysates were prepared by homogenization in RIPA buffer (150 mM NaCl, 50 mM Tris-Cl, pH8, 1% NP40, 0,5% deoxycholic acid, 0.1% SDS, 5% glycerol, 2 mM CaCl2, 10 mM EGTA) including protease inhibitors (P8340, Sigma-Aldrich) on ice. NuPAGE 4–12% bis Tris Zoom Gels (Invitrogen) were loaded with 2–10 µg of protein per well. SDS page gels were transferred onto nitrocellulose membranes (Amersham Bioscience), followed by reversible Ponceau S staining (P7170 Sigma St Louis) to control for loading and protein transfer. The membrane was blocked overnight in 5% skimmed milk in TBST (7.7 mM Tris, 150 mM SoNaCl, 0.5% Tween20) or Roti-Block (carl Roth GmbH), and incubated with the indicated antibody diluted in 2% skimmed milk or in Roti-Block or in 1% BSA in PBS. ECL or SuperSignal West Femto Maximum Sensitivity Substrate (Thermo Scientific) was used for detection in a Las-3000 Fujifilm machine.

List of primary antibodies (all polyclonal rabbit) anti-podocin (1∶2500, P0372 Sigma-Aldrich), anti-caveolin1 (1∶500, sc894, Santa Cruz Biotechnology), anti-claudin1 (1∶500, ab15098-500, Abcam), anti-pax2 (1∶500, 716000, Invitrogen), anti-WT1 (1∶500, sc192, Santa Cruz Biotechnology) and the monoclonal Golden Syrian Hamster anti-podoplanin (1∶500, NB600-1015, Novus Biologicals). Peroxidase labeled goat anti rabbit (Q0303 Vector Laboratories Burlingame, CA, USA) was used as secondary antibody (1∶10000) and streptavidin, horseradish peroxidase (1∶10000, SA-5004 Vector Laboratories Burlingame, CA, USA) as Biotin-avidin system.

### Immunohistochemistry

Paraffin sections were deparaffinized and blocked for endogenous avidin/biotin (Avidin/Biotin Blocking Kit, Vector Laboratories, Burlingame, CA) and peroxidase activity (3% H_2_O_2_). Subsequently, the sections were incubated with rabbit polyclonal anti-WT1 antibody (180) (1∶100, sc846, Santa Cruz Biotechnology, used for immunohistochemistry on mouse kidney sections) or anti-WT1 antiserum (C-19) (1∶100, sc192, Santa Cruz Biotechnology, CA, used for immunoblotting, IF of primary cells and for immunohistochemistry on rat and human kidney sections). As a secondary antibody, a biotinylated goat anti-rabbit was used (Vector Laboratories). Detection was carried out with Vectastain ABC Kit (Vector Laboratories) with the use of peroxidase as a label and 3-amino-9-ethylcarbazole as a substrate. Images were acquired using an Olympus BX 41 microscope and AnalySIS software.

### Video microscopy

The glomeruli were plated in glass bottom dishes (Willco-dish GWSt-5040) for four days. Phase contrast and epifluorescence images were acquired using an Axiovert 200 microscope (Carl Zeiss) equipped with a Plan-Neofluar 10×/0.30 numerical aperture objective. Images were recorded with a cooled, back-illuminated charge-coupled device camera (Cascade 512B; Princeton Instruments, Trenton, NJ) driven by IPLab Spectrum software (Scanalytics, Fairfax, USA). Digital handling of the images was done using IPLab Spectrum, ImageJ and Adobe Photoshop 8.0 (Adobe Systems, USA). The Delay between frames was 5 minutes. Phase contrast exposure time was 500 msec and fluorescence exposure time was 500 or 750 msec. The video shows 15 frames per second, so that 1 sec. corresponds to 75 min. capture time.

## Supporting Information

File S1 Table A. “Podocyte-specific genes”: List of differentially regulated transcripts, for which the expression in EGM-MV and RPMI did not differ by a factor of 2 or more in podocytes as well as in PECs, calculated as podocyte/PEC ratio. Only ratios of >4 were included and sorted by ratio. Table B. “PEC-specific genes”: PEC/podocyte ratio, selected and sorted as described above.
(DOC)

Video S1 After FACsorting, cultured parietal cells were filmed over a period of 70 hrs. (1 frame per 5 minutes). Cells can be seen transitioning between spindel-shaped and polygonal morphologies and undergoing complete cellular divisions. At the end of the aquisition, cells were fixed and stained for beta-gal confirming that all cells were derived from parietal cells (not shown).
(MOV)
