# Supplementary material for: Primary Cultures of Glomerular Parietal Epithelial Cells or Podocytes with Proven Origin
Source: PLoS One. 2012 Apr 18;7(4):e34907. doi: 10.1371/journal.pone.0034907 (PMC3329559; doi:10.1371/journal.pone.0034907)
Supplement: File S1 — Table A. “Podocyte-specific genes”: List of differentially regulated transcripts, for which the expression in EGM-MV and RPMI did not differ by a factor of 2 or more in podocytes as well as in PECs, calculated as podocyte/PEC ratio. Only ratios of >4 were included and sorted by ratio. Table B. “PEC-specific genes”: PEC/podocyte ratio, selected and sorted as described above. (DOC) [file pone.0034907.s001.doc]

**File S1**

**Table A, „Podocyte-specific“ transcripts**

| Gene symbol | Description | Pod/PEC ratio | *P*-value |
| --- | --- | --- | --- |
| Aldh1a1 | aldehyde dehydrogenase family 1, subfamily A1 Gene [Source:MGI (curated);Acc:MGI:1353450] | 60,1 | 2,0E-34 |
| Clic5 | chloride intracellular channel 5 Gene [Source:MGI Symbol;Acc:MGI:1917912] | 50,3 | 1,0E-85 |
| Sulf1 | sulfatase 1 Gene [Source:MGI Symbol;Acc:MGI:2138563] | 49,4 | 1,0E-46 |
| Efemp1 | epidermal growth factor-containing fibulin-like extracellular matrix protein 1 Gene [Source:MGI (curated);Acc:MGI:1339998] | 23,7 | 2,0E-89 |
| 4833424O15Rik | RIKEN cDNA 4833424O15 gene Gene [Source:MGI Symbol;Acc:MGI:1923019] | 19,5 | 2,0E-50 |
| Bmper | BMP-binding endothelial regulator Gene [Source:MGI Symbol;Acc:MGI:1920480] | 16,8 | 2,0E-29 |
| Cercam | cerebral endothelial cell adhesion molecule Gene [Source:MGI (curated);Acc:MGI:2139134] | 15,1 | 7,0E-36 |
| Rcn3 | reticulocalbin 3, EF-hand calcium binding domain Gene [Source:MGI (curated);Acc:MGI:1277122] | 15,0 | 4,0E-12 |
| Cox6b2 | cytochrome c oxidase subunit VIb polypeptide 2 Gene [Source:MGI Symbol;Acc:MGI:3044182] | 14,1 | 2,0E-13 |
| Gpc3 | glypican 3 Gene [Source:MGI (curated);Acc:MGI:104903] | 13,5 | 5,0E-40 |
| Pde3a | phosphodiesterase 3A, cGMP inhibited Gene [Source:MGI Symbol;Acc:MGI:1860764] | 13,3 | 6,0E-39 |
| Mmp23 | matrix metallopeptidase 23 Gene [Source:MGI (curated);Acc:MGI:1347361] | 13,0 | 6,0E-36 |
| Lgals9 | lectin, galactose binding, soluble 9 Gene [Source:MGI (curated);Acc:MGI:109496] | 12,2 | 8,0E-15 |
| St3gal6 | ST3 beta-galactoside alpha-2,3-sialyltransferase 6 Gene [Source:MGI (curated);Acc:MGI:1888707] | 11,9 | 1,0E-40 |
| Pdgfrl | platelet-derived growth factor receptor-like Gene [Source:MGI (curated);Acc:MGI:1916047] | 11,8 | 4,0E-28 |
| Mustn1 | musculoskeletal, embryonic nuclear protein 1 Gene [Source:MGI Symbol;Acc:MGI:1913425] | 11,7 | 2,0E-08 |
| Ctsz | cathepsin Z Gene [Source:MGI (curated);Acc:MGI:1891190] | 11,3 | 3,0E-38 |
| Bgn | biglycan Gene [Source:MGI (curated);Acc:MGI:88158] | 11,2 | 1,5E-08 |
| Abat | 4-aminobutyrate aminotransferase Gene [Source:MGI (curated);Acc:MGI:2443582] | 11,2 | 2,0E-35 |
| Aff2 | AF4/FMR2 family, member 2 Gene [Source:MGI (curated);Acc:MGI:1202294] | 11,2 | 6,0E-18 |
| Arhgap28 | Rho GTPase activating protein 28 Gene [Source:MGI Symbol;Acc:MGI:2147003] | 11,1 | 6,0E-52 |
| Col1a2 | collagen, type I, alpha 2 Gene [Source:MGI (curated);Acc:MGI:88468] | 10,8 | 1,0E-61 |
| Tceal1 | transcription elongation factor A (SII)-like 1 Gene [Source:MGI (curated);Acc:MGI:2385317] | 9,9 | 1,0E-10 |
| Prkar1b | protein kinase, cAMP dependent regulatory, type I beta Gene [Source:MGI (curated);Acc:MGI:97759] | 9,6 | 4,3E-07 |
| Mertk | c-mer proto-oncogene tyrosine kinase Gene [Source:MGI (curated);Acc:MGI:96965] | 9,5 | 5,0E-47 |
| Nid1 | predicted gene 2399 Gene [Source:MGI Symbol;Acc:MGI:3780567] | 9,4 | 3,0E-19 |
| Rarres1 | retinoic acid receptor responder (tazarotene induced) 1 Gene [Source:MGI Symbol;Acc:MGI:1924461] | 8,5 | 2,0E-13 |
| Accn3 | amiloride-sensitive cation channel 3 Gene [Source:MGI Symbol;Acc:MGI:2159339] | 8,4 | 4,0E-17 |
| Acot11 | acyl-CoA thioesterase 11 Gene [Source:MGI (curated);Acc:MGI:1913736] | 8,3 | 2,0E-27 |
| Pdpn | podoplanin Gene [Source:MGI (curated);Acc:MGI:103098] | 7,8 | 4,0E-18 |
| C1s | complement component 1, s subcomponent Gene [Source:MGI Symbol;Acc:MGI:1355312] | 7,4 | 1,7E-08 |
| Rapgef3 | Rap guanine nucleotide exchange factor (GEF) 3 Gene [Source:MGI (curated);Acc:MGI:2441741] | 7,4 | 6,0E-23 |
| C130021I20Rik | Riken cDNA C130021I20 gene Gene [Source:MGI Symbol;Acc:MGI:3639863] | 7,3 | 8,0E-16 |
| Tmem45a | transmembrane protein 45a Gene [Source:MGI (curated);Acc:MGI:1913122] | 7,2 | 1,0E-30 |
| Nupr1 | nuclear protein 1 Gene [Source:MGI (curated);Acc:MGI:1891834] | 7,0 | 5,0E-19 |
| Lass4 | LAG1 homolog, ceramide synthase 4 Gene [Source:MGI Symbol;Acc:MGI:1914510] | 7,0 | 1,0E-14 |
| Cd55 | CD55 antigen Gene [Source:MGI (curated);Acc:MGI:104850] | 6,7 | 1,0E-10 |
| Arhgap20 | Rho GTPase activating protein 20 Gene [Source:MGI (curated);Acc:MGI:2445175] | 6,6 | 3,0E-28 |
| Mrc2 | mannose receptor, C type 2 Gene [Source:MGI (curated);Acc:MGI:107818] | 6,6 | 2,0E-23 |
| Zfp385b | zinc finger protein 385B Gene [Source:MGI (curated);Acc:MGI:2444734] | 6,5 | 9,0E-34 |
| Unc5c | unc-5 homolog C (C. elegans) Gene [Source:MGI (curated);Acc:MGI:1095412] | 6,3 | 8,0E-50 |
| Ptgis | prostaglandin I2 (prostacyclin) synthase Gene [Source:MGI (curated);Acc:MGI:1097156] | 6,2 | 2,0E-26 |
| Nbl1 | neuroblastoma, suppression of tumorigenicity 1 Gene [Source:MGI (curated);Acc:MGI:104591] | 6,1 | 1,0E-18 |
| Lsp1 | lymphocyte specific 1 Gene [Source:MGI (curated);Acc:MGI:96832] | 6,1 | 4,0E-25 |
| Nlgn1 | neuroligin 1 Gene [Source:MGI Symbol;Acc:MGI:2179435] | 5,9 | 2,0E-28 |
| Lrp1 | low density lipoprotein receptor-related protein 1 Gene [Source:MGI (curated);Acc:MGI:96828] | 5,9 | 7,0E-56 |
| Hspa12a | heat shock protein 12A Gene [Source:MGI Symbol;Acc:MGI:1920692] | 5,8 | 8,0E-20 |
| Unc93b1 | unc-93 homolog B1 (C. elegans) Gene [Source:MGI Symbol;Acc:MGI:1859307] | 5,7 | 2,0E-15 |
| Gstm4 | glutathione S-transferase, mu 4 Gene [Source:MGI (curated);Acc:MGI:95862] | 5,7 | 1,2E-08 |
| Galnt10 | UDP-N-acetyl-alpha-D-galactosamine:polypeptide N-acetylgalactosaminyltransferase 10 Gene [Source:MGI (curated);Acc:MGI:1890480] | 5,7 | 1,0E-16 |
| Lpl | lipoprotein lipase Gene [Source:MGI Symbol;Acc:MGI:96820] | 5,7 | 1,0E-18 |
| Emilin1 | elastin microfibril interfacer 1 Gene [Source:MGI Symbol;Acc:MGI:1926189] | 5,7 | 5,0E-26 |
| Plekhg1 | pleckstrin homology domain containing, family G (with RhoGef domain) member 1 Gene [Source:MGI (curated);Acc:MGI:2676551] | 5,6 | 2,0E-17 |
| A430107O13Rik | RIKEN cDNA A430107O13 gene Gene [Source:MGI Symbol;Acc:MGI:2444814] | 5,6 | 4,0E-17 |
| Fstl3 | follistatin-like 3 Gene [Source:MGI Symbol;Acc:MGI:1890391] | 5,5 | 3,0E-16 |
| Rtn2 | reticulon 2 (Z-band associated protein) Gene [Source:MGI Symbol;Acc:MGI:107612] | 5,4 | 3,0E-11 |
| Reck | reversion-inducing-cysteine-rich protein with kazal motifs Gene [Source:MGI (curated);Acc:MGI:1855698] | 5,4 | 1,0E-25 |
| Itgb5 | integrin beta 5 Gene [Source:MGI (curated);Acc:MGI:96614] | 5,4 | 2,0E-23 |
| Zfp605 | zinc finger protein 605 Gene [Source:MGI (curated);Acc:MGI:2444933] | 5,4 | 1,0E-13 |
| Gata2 | GATA binding protein 2 Gene [Source:MGI Symbol;Acc:MGI:95662] | 5,3 | 9,0E-21 |
| Fkbp10 | FK506 binding protein 10 Gene [Source:MGI (curated);Acc:MGI:104769] | 5,3 | 1,0E-18 |
| Fam18a | family with sequence similarity 18, member A Gene [Source:MGI (curated);Acc:MGI:3665441] | 5,3 | 2,9E-07 |
| Lynx1 | Ly6/neurotoxin 1 Gene [Source:MGI Symbol;Acc:MGI:1345180] | 5,3 | 3,8E-06 |
| Bcam | basal cell adhesion molecule Gene [Source:MGI (curated);Acc:MGI:1929940] | 5,2 | 4,0E-26 |
| Slc5a7 | solute carrier family 5 (choline transporter), member 7 Gene [Source:MGI Symbol;Acc:MGI:1927126] | 5,2 | 8,0E-28 |
| Sorbs3 | sorbin and SH3 domain containing 3 Gene [Source:MGI Symbol;Acc:MGI:700013] | 5,1 | 2,0E-24 |
| Pdlim2 | PDZ and LIM domain 2 Gene [Source:MGI (curated);Acc:MGI:2384850] | 4,9 | 3,0E-18 |
| H2-T23 | histocompatibility 2, T region locus 23 Gene [Source:MGI (curated);Acc:MGI:95957] | 4,9 | 1,6E-06 |
| Jam2 | junction adhesion molecule 2 Gene [Source:MGI (curated);Acc:MGI:1933820] | 4,9 | 2,0E-12 |
| Speg | SPEG complex locus Gene [Source:MGI (curated);Acc:MGI:109282] | 4,8 | 2,0E-27 |
| Cyp27a1 | cytochrome P450, family 27, subfamily a, polypeptide 1 Gene [Source:MGI Symbol;Acc:MGI:88594] | 4,8 | 1,0E-21 |
| Gpx7 | glutathione peroxidase 7 Gene [Source:MGI (curated);Acc:MGI:1914555] | 4,8 | 7,0E-15 |
| Serpina3h | serine (or cysteine) peptidase inhibitor, clade A, member 3H Gene [Source:MGI Symbol;Acc:MGI:2182839] | 4,8 | 4,0E-10 |
| Qsox1 | quiescin Q6 sulfhydryl oxidase 1 Gene [Source:MGI (curated);Acc:MGI:1330818] | 4,8 | 3,0E-12 |
| Stim1 | stromal interaction molecule 1 Gene [Source:MGI Symbol;Acc:MGI:107476] | 4,7 | 4,0E-13 |
| Flrt2 | fibronectin leucine rich transmembrane protein 2 Gene [Source:MGI (curated);Acc:MGI:3603594] | 4,7 | 3,0E-17 |
| Zbtb7c | zinc finger and BTB domain containing 7C Gene [Source:MGI Symbol;Acc:MGI:2443302] | 4,7 | 3,0E-17 |
| Tm6sf1 | transmembrane 6 superfamily member 1 Gene [Source:MGI (curated);Acc:MGI:1933209] | 4,6 | 8,0E-27 |
| Tmem108 | transmembrane protein 108 Gene [Source:MGI Symbol;Acc:MGI:1932411] | 4,6 | 3,0E-17 |
| Gstk1 | glutathione S-transferase kappa 1 Gene [Source:MGI Symbol;Acc:MGI:1923513] | 4,5 | 1,0E-16 |
| H2-D1 | histocompatibility 2, D region Gene [Source:MGI Symbol;Acc:MGI:95912] | 4,5 | 1,0E-12 |
| Synpo | synaptopodin Gene [Source:MGI (curated);Acc:MGI:1099446] | 4,5 | 1,6E-09 |
| Raver2 | ribonucleoprotein, PTB-binding 2 Gene [Source:MGI (curated);Acc:MGI:2443623] | 4,5 | 8,0E-39 |
| Ptprd | protein tyrosine phosphatase, receptor type, D Gene [Source:MGI (curated);Acc:MGI:97812] | 4,4 | 7,0E-31 |
| Colec12 | collectin sub-family member 12 Gene [Source:MGI Symbol;Acc:MGI:2152907] | 4,3 | 1,4E-08 |
| Lmx1b | LIM homeobox transcription factor 1 beta Gene [Source:MGI (curated);Acc:MGI:1100513] | 4,3 | 3,0E-21 |
| Mtap1a | microtubule-associated protein 1 A Gene [Source:MGI (curated);Acc:MGI:1306776] | 4,3 | 5,0E-17 |
| Adm | adrenomedullin Gene [Source:MGI Symbol;Acc:MGI:108058] | 4,3 | 1,6E-05 |
| H6pd | hexose-6-phosphate dehydrogenase (glucose 1-dehydrogenase) Gene [Source:MGI (curated);Acc:MGI:2140356] | 4,3 | 9,0E-15 |
| Slc39a8 | solute carrier family 39 (metal ion transporter), member 8 Gene [Source:MGI (curated);Acc:MGI:1914797] | 4,2 | 3,0E-11 |
| Bok | BCL2-related ovarian killer protein Gene [Source:MGI Symbol;Acc:MGI:1858494] | 4,2 | 2,0E-14 |
| Pros1 | protein S (alpha) Gene [Source:MGI (curated);Acc:MGI:1095733] | 4,1 | 5,0E-20 |
| Fam49a | family with sequence similarity 49, member A Gene [Source:MGI Symbol;Acc:MGI:1261783] | 4,1 | 6,0E-10 |
| Gng2 | guanine nucleotide binding protein (G protein), gamma 2 Gene [Source:MGI Symbol;Acc:MGI:102705] | 4,1 | 8,0E-13 |
| Golim4 | golgi integral membrane protein 4 Gene [Source:MGI (curated);Acc:MGI:1920374] | 4,1 | 3,0E-17 |
| Arhgef16 | Rho guanine nucleotide exchange factor (GEF) 16 Gene [Source:MGI (curated);Acc:MGI:2446219] | 4,1 | 3,0E-15 |
| Loxl2 | lysyl oxidase-like 2 Gene [Source:MGI Symbol;Acc:MGI:2137913] | 4,0 | 7,2E-09 |
| Chst1 | carbohydrate (keratan sulfate Gal-6) sulfotransferase 1 Gene [Source:MGI (curated);Acc:MGI:1924219] | 4,0 | 6,0E-12 |
| Mtss1 | metastasis suppressor 1 Gene [Source:MGI Symbol;Acc:MGI:2384818] | 4,0 | 2,0E-23 |

**Table B, „PEC-specific“ transcripts**

| **Gene symbol** | **Description** | **PEC/Pod**  **ratio** | ***P*-value** |
| --- | --- | --- | --- |
| Car2 | carbonic anhydrase 2 Gene [Source:MGI Symbol;Acc:MGI:88269] | 29,1 | 2,0E-19 |
| Tmem171 | transmembrane protein 171 Gene [Source:MGI (curated);Acc:MGI:2685751] | 22,8 | 7,0E-54 |
| Gc | group specific component Gene [Source:MGI Symbol;Acc:MGI:95669] | 17,9 | 8,0E-21 |
| Fermt1 | fermitin family homolog 1 (Drosophila) Gene [Source:MGI (curated);Acc:MGI:2443583] | 14,2 | 2,0E-13 |
| Igsf11 | immunoglobulin superfamily, member 11 Gene [Source:MGI (curated);Acc:MGI:2388477] | 13,5 | 3,0E-20 |
| Mboat2 | membrane bound O-acyltransferase domain containing 2 Gene [Source:MGI Symbol;Acc:MGI:1914466] | 13,2 | 2,0E-30 |
| Hey1 | hairy/enhancer-of-split related with YRPW motif 1 Gene [Source:MGI Symbol;Acc:MGI:1341800] | 12,8 | 6,0E-21 |
| F2rl1 | coagulation factor II (thrombin) receptor-like 1 Gene [Source:MGI Symbol;Acc:MGI:101910] | 11,1 | 4,0E-43 |
| Ank3 | ankyrin 3, epithelial Gene [Source:MGI Symbol;Acc:MGI:88026] | 10,7 | 8,0E-50 |
| Tcfcp2l1 | transcription factor CP2-like 1 Gene [Source:MGI Symbol;Acc:MGI:2444691] | 9,7 | 5,0E-32 |
| Trpm6 | transient receptor potential cation channel, subfamily M, member 6 Gene [Source:MGI Symbol;Acc:MGI:2675603] | 9,7 | 8,0E-41 |
| Lad1 | ladinin Gene [Source:MGI Symbol;Acc:MGI:109343] | 9,4 | 4,0E-14 |
| Eya2 | eyes absent 2 homolog (Drosophila) Gene [Source:MGI (curated);Acc:MGI:109341] | 8,9 | 4,0E-37 |
| A330021E22Rik | RIKEN cDNA A330021E22 gene Gene [Source:MGI Symbol;Acc:MGI:2443778] | 8,2 | 2,0E-28 |
| Clca4 | chloride channel calcium activated 4 Gene [Source:MGI Symbol;Acc:MGI:2181989] | 8,0 | 1,0E-19 |
| Mia2 | melanoma inhibitory activity 2 Gene [Source:MGI (curated);Acc:MGI:2159614] | 7,9 | 7,0E-27 |
| Scin | scinderin Gene [Source:MGI (curated);Acc:MGI:1306794] | 7,8 | 3,0E-34 |
| Tbx15 | T-box 15 Gene [Source:MGI (curated);Acc:MGI:1277234] | 7,7 | 4,0E-37 |
| Acp1 | acid phosphatase 1, soluble Gene [Source:MGI Symbol;Acc:MGI:87881] | 7,3 | 3,2E-06 |
| Slco2a1 | solute carrier organic anion transporter family, member 2a1 Gene [Source:MGI Symbol;Acc:MGI:1346021] | 6,9 | 4,0E-25 |
| Cdk18 | cyclin-dependent kinase 18 Gene [Source:MGI Symbol;Acc:MGI:97518] | 6,5 | 4,0E-19 |
| Postn | periostin, osteoblast specific factor Gene [Source:MGI (curated);Acc:MGI:1926321] | 6,5 | 2,0E-46 |
| Pde8a | phosphodiesterase 8A Gene [Source:MGI (curated);Acc:MGI:1277116] | 6,5 | 2,0E-24 |
| Ccdc141 | coiled-coil domain containing 141 Gene [Source:MGI (curated);Acc:MGI:1919735] | 6,4 | 1,0E-57 |
| Hunk | hormonally upregulated Neu-associated kinase Gene [Source:MGI Symbol;Acc:MGI:1347352] | 6,3 | 3,0E-40 |
| Ntm | neurotrimin Gene [Source:MGI (curated);Acc:MGI:2446259] | 6,2 | 6,0E-09 |
| Edn1 | endothelin 1 Gene [Source:MGI Symbol;Acc:MGI:95283] | 6,1 | 2,0E-33 |
| Fam176a | family with sequence similarity 176, member A Gene [Source:MGI (curated);Acc:MGI:2385247] | 5,8 | 6,0E-20 |
| Lrrn1 | leucine rich repeat protein 1, neuronal Gene [Source:MGI Symbol;Acc:MGI:106038] | 5,5 | 1,1E-07 |
| B4galt6 | UDP-Gal:betaGlcNAc beta 1,4-galactosyltransferase, polypeptide 6 Gene [Source:MGI Symbol;Acc:MGI:1928380] | 5,4 | 6,0E-28 |
| Ivns1abp | influenza virus NS1A binding protein Gene [Source:MGI Symbol;Acc:MGI:2152389] | 5,3 | 1,0E-20 |
| Clu | clusterin Gene [Source:MGI (curated);Acc:MGI:88423] | 5,3 | 3,9E-07 |
| Cdh3 | cadherin 3 Gene [Source:MGI Symbol;Acc:MGI:88356] | 5,3 | 1,4E-05 |
| Gpnmb | glycoprotein (transmembrane) nmb Gene [Source:MGI Symbol;Acc:MGI:1934765] | 5,2 | 4,0E-32 |
| Plch1 | phospholipase C, eta 1 Gene [Source:MGI Symbol;Acc:MGI:2683547] | 5,1 | 4,8E-08 |
| Epha7 | Eph receptor A7 Gene [Source:MGI (curated);Acc:MGI:95276] | 5,0 | 4,0E-13 |
| Dysf | dysferlin Gene [Source:MGI (curated);Acc:MGI:1349385] | 5,0 | 3,0E-41 |
| Enpp1 | ectonucleotide pyrophosphatase/phosphodiesterase 1 Gene [Source:MGI (curated);Acc:MGI:97370] | 4,9 | 1,8E-05 |
| Rabl2 | RAB, member of RAS oncogene family-like 2A Gene [Source:MGI Symbol;Acc:MGI:1915958] | 4,9 | 1,0E-17 |
| Ccdc109b | coiled-coil domain containing 109B Gene [Source:MGI (curated);Acc:MGI:1914065] | 4,8 | 7,0E-28 |
| Arhgef3 | Rho guanine nucleotide exchange factor (GEF) 3 Gene [Source:MGI Symbol;Acc:MGI:1918954] | 4,7 | 1,0E-19 |
| Tuft1 | tuftelin 1 Gene [Source:MGI (curated);Acc:MGI:109572] | 4,7 | 2,1E-09 |
| 4930547N16Rik | RIKEN cDNA 4930547N16 gene Gene [Source:MGI Symbol;Acc:MGI:1922567] | 4,6 | 4,0E-30 |
| Vldlr | very low density lipoprotein receptor Gene [Source:MGI Symbol;Acc:MGI:98935] | 4,5 | 5,0E-15 |
| Abcc4 | ATP-binding cassette, sub-family C (CFTR/MRP), member 4 Gene [Source:MGI Symbol;Acc:MGI:2443111] | 4,5 | 3,0E-12 |
| Gpr126 | G protein-coupled receptor 126 Gene [Source:MGI Symbol;Acc:MGI:1916151] | 4,4 | 3,0E-21 |
| Samd9l | sterile alpha motif domain containing 9-like Gene [Source:MGI (curated);Acc:MGI:1343184] | 4,4 | 6,0E-13 |
| Il1rl1 | interleukin 1 receptor-like 1 Gene [Source:MGI Symbol;Acc:MGI:98427] | 4,4 | 4,0E-23 |
| Aim1 | absent in melanoma 1 Gene [Source:MGI Symbol;Acc:MGI:109544] | 4,3 | 9,0E-20 |
| Ly75 | lymphocyte antigen 75 Gene [Source:MGI (curated);Acc:MGI:106662] | 4,3 | 2,0E-32 |
| Slc40a1 | solute carrier family 40 (iron-regulated transporter), member 1 Gene [Source:MGI Symbol;Acc:MGI:1315204] | 4,2 | 1,8E-07 |
| Mecom | MDS1 and EVI1 complex locus Gene [Source:MGI Symbol;Acc:MGI:95457] | 4,1 | 1,0E-31 |
| Ahr | aryl-hydrocarbon receptor Gene [Source:MGI Symbol;Acc:MGI:105043] | 4,1 | 4,1E-08 |
| Pcyt1b | phosphate cytidylyltransferase 1, choline, beta isoform Gene [Source:MGI (curated);Acc:MGI:2147987] | 4,1 | 5,0E-16 |
| Ppl | periplakin Gene [Source:MGI Symbol;Acc:MGI:1194898] | 4,1 | 8,0E-21 |
| 4632434I11Rik | RIKEN cDNA 4632434I11 gene Gene [Source:MGI Symbol;Acc:MGI:1921291] | 4,0 | 7,0E-17 |
